# Does sense of work gain predict team creativity? The mediating effect of leader-member exchange and the moderating effect of work smoothness

**DOI:** 10.3389/fpsyg.2023.1043376

**Published:** 2023-02-21

**Authors:** Kun Shi, Zhifan Zhang, Hua Zhang

**Affiliations:** Faculty of Psychology, Southwest University, Chongqing, China

**Keywords:** work smoothness, team creativity, LMX, the sense of work gain, management

## Abstract

In the present study, we examined the link between the sense of work gain and team creativity and explored the mediating and moderating roles of Leader-member exchange (LMX) and work smoothness on it. The results of this study A moderated mediation model was constructed based on 484 valid samples from an on-line survey of a human resource company, revealed that the sense of work gain can positively predict team creativity, and LMX mediates the associations of the sense of work gain and team creativity. Moreover, work smoothness emerged as a significant moderator can moderate the associations between sense of work gain and team creativity, as well as moderating the relationship between LMX and team creativity. The findings provide a theoretical guidance for leaders and HR professionals who want to increase employee initiative and motivation.

## 1. Introduction

Innovation and creativity have always been regarded as important themes of social development. The Xinhua News Agency (2020) reported that the Chinese government adheres to in-depth implementation of an innovation-driven strategy of developing the country through science and education, and is accelerating the construction of a scientific and technological power. With the support of national policies, enterprises are actively promoting innovation and creativity to enhance competitiveness and achieve better out comes.

Creativity is distinct from, but closely related to, innovation; it is a precondition of innovation (Hughes et al., 2018). Team creativity, the key motivation for an organization to achieve innovation performance and competitive advantage (Ren et al., 2022), refers to the collaboration of team members to create products, ideas, and procedures that are valuable, useful, and innovative in a complex social system (Woodman et al., 1993). It has become a hot topic in both theoretical and practical circles.

In the past 10 years, research on team creativity in China has become active, focusing on the effect of leadership styles (Peng et al., 2020; Li et al., 2021; Yang et al., 2021) and leader–member exchange (LMX) (Wang et al., 2009; Cai et al., 2013) on team creativity from the perspective of leadership. Despite these advances, few studies have discussed the mechanisms behind these different effects from the employees’ perspective. Ren et al. (2022) pointed out that future research should be based on China’s unique cultural characteristics and value systems. Additionally, it is necessary to combine different levels of creativity and related factors (individuals, teams, and organizations). Following these expectations, the present study aimed to investigate the relationship between the local individual-level variable of employees’ sense of work gain (Zhu and Liu, 2020) and team creativity. Furthermore, we used LMX and work smoothness (Jin et al., 2021) to explore the mechanism linking sense of work gain and team creativity.

## 2. Literature review and hypotheses

### 2.1. Sense of work gain and team creativity

Sense of work gain is an individual’s comprehensive feeling of actual reward from work and the value of its realization. It includes four aspects: job dignity, pay satisfaction, ability improvement, and sense of career vision (Zhu and Liu, 2020). As a native Chinese concept, it originally refers to the satisfaction generated by obtaining certain benefits—reflecting people’s desire to develop fairness and justice, and achieve their desired social needs for a better life (Cao and Li, 2017). Although there is a lack of empirical research on the relationship between sense of work gain and team creativity, previous studies have reported that the four aspects of sense of work gain are closely related to the factors affecting team creativity (Wright and Huang, 2012; Zhu and Liu, 2020). Therefore, we proposed that sense of work gain can affect team creativity.

Zhu and Liu (2020) conducted a nomological network analysis which demonstrated that sense of work gain is the basis of job well-being, and that job acquisition can positively predict employees’ job well-being. Sense of work gain not only gives employees more positive emotional experiences (Bakker, 2015) but also increases their job satisfaction (Wright and Huang, 2012). Furthermore, positive emotional experiences (Grawitch et al., 2003) and high job satisfaction are important factors that stimulate creativity (Wright and Huang, 2012).

In an atmosphere of positive emotions and high job satisfaction, employees create a positive group-working environment. According to the emotional information proposition theory (Ashby et al., 1999), when an individual’s emotions are stimulated, they can mobilize the relevant cognitive functions to start working, which then begin to activate creative cognitive resources (Deng and Huang, 2010). Consequently, in a positive group environment, employees within the team generate more cognitive exchanges to promote team creativity.

Considering the above, the following hypothesis was proposed:

*H1*: Sense of work gain positively predicts team creativity.

### 2.2. Leader-member exchange and team creativity

In Chinese culture, the relationship between leaders and employees has a long history, and it continues to have a significant influence on the attitudes and behaviors of people in modern organizations (Zhang et al., 2020). Thus, in discussing the impact of employees’ sense of work gain on team creativity, the relationship between leadership and employees can play an important role in the organizational context as critical social capital (Chen et al., 2013). LMX refers to the exchange relationship established by leadership and employees at work (Graen and Cashman, 1975), wherein leaders and employees develop unique relationships (Graen and Uhl-Bien, 1995) that consequently produce different leadership-member exchange relationship qualities (Rockstuhl et al., 2012; Zhong, 2018). From the perspectives of the social exchange theory and social comparison theory, some studies have concluded that LMX differentiation can predict team creativity (Chen and Liu, 2020; He et al., 2021).

However, LMX differentiation is not equivalent to LMX. LMX reflects the reciprocal process of bilateral relations between leaders and team members (Pei et al., 2013), whereas LMX quality is the result of leadership interactions with employees. The theory of regulating focus holds that leaders can constantly stimulate their work attitudes and behaviors by adjusting the focus of their subordinates’ situational patterns (Li and Shang, 2011). Thus, LMX can positively predict employee creativity (Tierney, 2015). Team creativity is generally regarded as an additive model (predicting average member creativity) or a disjunctive model (predicting highest member creativity; Yuan et al., 2022). In this study, we propose that LMX can predict the output of a team’s creativity.

How does LMX predict the output of a team’s creativity? In a team, more communication between leaders and employees can improve mutual understanding, correct communication errors, reduce communication barriers, more easily produce positive work outcomes (Ilies et al., 2007), and establish good social and emotional connections between team members to make them feel safe in the work environment (Spreitzer et al., 2010). This is considered to be an important factor in promoting creative output (Scott and Bruce, 1994).

Considering the above, the following hypothesis was proposed:

*H2*: LMX positively predicts team creativity.

### 2.3. Mediating effect of leader-member exchange

Sense of work gain is an individual’s comprehensive feeling regarding their actual pay return and value realization (including the degree to which employee work achievements are recognized by others in the organization), the material basis of an employee’s psychological contract, and the employee’s growing work support environment as well as training and growth opportunities or platforms provided by the organization (Zhu and Liu, 2020).

As a dynamic development process, LMX involves both material and psychological exchanges (Lv, 2013). For material acquisition, a material reward can satisfy the material basis of employees’ psychological contract in order to improve their working conditions and foster higher job output that attracts more recognition from leadership. Therefore, a good LMX relationship is a channel through which leaders allocate organizational resources, assign task-related benefits, and provide psychological support to their subordinates (Graen and Uhl-Bien, 1995).

Previous studies have shown that employees’ sense of job dignity, compensation satisfaction, ability to improve, and career aspirations can influence leadership’s evaluation of employees and employees’ perceptions of LMX quality.

On the one hand, for psychological gain, employees are recognized by the leadership; in the process of affirming employees, verbal encouragement and emotional care from leaders can narrow the distance between superiors and subordinates, promote effective interaction between them, enhance trust and belonging, and improve subordinates’ work and learning enthusiasm (Wang et al., 2017). This establishes a higher LMX and creates a good team atmosphere. On the other hand, employees should in turn recognize leadership behavior. Walumbwa and Hartnell (2011) found that employees’ perception of their superiors’ identity is positively correlated with self-efficacy, which in turn has a positive relationship with employee performance—including creativity and innovation. Employees’ recognition of leadership is positively correlated with employee creativity, which in turn promotes team creativity. Sense of work gain can enhance employees’ leadership recognition through their self-efficacy and help to narrow the psychological distance between leaders and employees (Gu et al., 2015). A good team atmosphere is imperative for team creativity (Li et al., 2018).

Considering the above, the following hypothesis was proposed:

*H3*: LMX acts as an intermediary between sense of work gain and team creativity.

### 2.4. Work smoothness plays a role in regulating sense of work gain and team creativity

Discussions about job alienation among different occupational groups are common; job alienation refers to the psychological state of separation between employees and work, caused by job situations that do not meet employee needs or expectations (Banaia et al., 2004). However, work does not always cause employees to feel dissatisfied. Getting help (from leaders or colleagues) when one feels stuck in the workplace not only improves job performance but also enhances feelings for one’s colleagues. Therefore, we included the concept of work smoothness in this study to discuss its positive effects.

Work smoothness refers to the positive subjective experience gained by getting help from leaders and colleagues to make one’s job run smoothly when work is stymied. The smooth progress of work can improve teamwork efficiency and team cohesion (Jin et al., 2021). In such a work environment, employees’ job performance is more likely to be recognized by colleagues or leaders. Consequently, employees’ sense of work gain increases significantly when working with teams that have high-level work smoothness, which facilitates mutual assistance among members, increases turnover and utilization of resources within the organization (Keshet et al., 1991), and promotes the process of future team internal innovation (Tsai et al., 2012). Moreover, as team members interact with each other, they enhance the level of task reflection amongst themselves, which can further improve the team’s creativity based on lessons learned from failures (Shin, 2014).

Considering the above, the following hypothesis was proposed:

*H4*: Work smoothness significantly enhances the impact of sense of work gain on team creativity.

### 2.5. Work smoothness plays a role in regulating leader-member exchange between team creativity

Work smoothness implies that when employees encounter obstacles in their work, they receive help from leaders or colleagues to make the work run smoothly and have a positive subjective experience, unlike the negative experiences produced by a sense of job alienation. Work smoothness can improve employee productivity (Jin et al., 2021) and foster positive emotions among employees, which subsequently has a positive impact on LMX quality and sense of work gain from work perception. Therefore, it is believed that work smoothness can not only play a regulatory role—affecting the role of sense of work gain in team creativity—but also affect the role of LMX in team creativity. As a process of interchange between leaders and members (Graen and Uhl-Bien, 1995), LMX improves the work smoothness of employees with the help of the leader so that their productivity increases significantly, which in turn leads to better leadership quality. By improving LMX quality, employees enjoy a higher level of trust, respect, and emotional support, and leaders allocate more tangible or intangible work resources to their subordinates (Chaudhry et al., 2020). Employees with more resources are more likely to attempt everyday tasks and gain respect, mission-related recognition, and interpersonal encouragement (Tierney et al., 1999; Tierney, 2008). This creates a virtuous circle, creating an atmosphere of mutual support and energy with a high degree of trust that helps promote team creativity (Wu and Zhang, 2017).

Considering the above, the following hypothesis was proposed:

*H5*: Work smoothness significantly enhances the impact of LMX on team creativity.

## 3. Methods

### 3.1. Samples and procedures

China Report Network (2021) reported that, at present, China’s small and medium-sized enterprises have the typical characteristics of “56,789,” contributing more than 50% of tax revenue, more than 60% of GDP, more than 70% of technological innovation, more than 80% of urban labor and employment, and more than 90% of the number of enterprises—these are the driving forces of the national economic and social development. Therefore, this study selected domestic Chinese small and medium-sized enterprises as the research subjects.

The data were collected using an online survey conducted by a human resources company in northern China. Their senior management agreed to collect data from nine of its branches, requested survey results, and consulting suggestions. Prior to the survey, the human resource managers helped notify and invite all eligible employees to participate in the survey. This company follows a flat management style, which means that there are clear standards and promotion channels for all employees, and that employee innovation is an essential element for business. Therefore, to avoid homogeneous deviation, we selected company branches in different areas of China (such as North and Northeast China) as the study subjects.

To ensure accurate assessment of the real data, all participants’ responses were anonymous. We shared guidelines before the start of the survey which explicitly stated that no personal information would be disclosed, including the measurement of sensitive information during the administration of the questionnaire, so that employees could more readily share their true perceptions. In addition, the questionnaires were web-based, and the researcher was the only observer allowed around participants to prevent any influence of social expectations on participant responses.

In total, 505 employees from different locations participated in our survey, of which 484 (95.8%) submitted valid questionnaires. Among the final valid sample, 41.9% were male and 58.1% were female. The age distribution of the subjects in this survey was 30.1 (±5.32). Regarding their educational background, 9.3% had an education below a junior college degree, 26.7% had a junior college degree, 57.2% had a bachelor’s degree, and 6.8% had a master’s degree or above. In terms of on-boarding time, the proportion of employees in the first year of employment was 14.7%, in 1–3 years was 36.8%, 3–5 years was 26.7%, and > 5 years was 21.9%.

### 3.2. Measurements

A 5-point Likert scale was used for assessing each item, with 1 indicating “strongly disagree” and 5 indicating “strongly agree.”

#### 3.2.1. Sense of work gain

This scale developed by Zhu and Liu (2020) consists of 13 items in total, including “my work has been affirmed by superiors,” “I get a reasonable salary,” “the work lets me master the job knowledge and skills,” and “I am engaged in work related to my career ideal.” (Cronbach’s *α* = 0.914), respectively. See the Appendix for the complete scale.

#### 3.2.2. Leader-member exchange

We measured the LMX relationship using the LMX-7 scale developed by Scandura et al. (1986); the items include “I think I have a good relationship with my immediate supervisor,” “my personal satisfaction with my current job, my direct supervisor knows it well,” and “I think my direct supervisor knows my job potential very well” with a total of 7 items. (Cronbach’s *α* = 0.859).

#### 3.2.3. Work smoothness

To assess the smoothness of employees’ work, we adopted a work alienation scale developed by Wang (2018). We removed two items from the original scale that do not explicitly relate to work smoothness—“At work, you cannot say what you think” and “At work, you cannot make your own choices” (Lee, 2019). The items included were “when there is a problem getting in the way of work, you cannot get the spiritual support of your boss,” “when you face a barrier to work, you cannot get effective help from your boss,” and “when you face a barrier, you cannot get effective help from your colleagues.” The scale used reverse scoring to measure the smoothness of an employee’s work and included a total of three items. (Cronbach’s *α* = 0.878).

#### 3.2.4. Team creativity

We translated a team-created power scale created by Shin and Zhou (2007) into Chinese using the standard translation procedures. The items include “our team always comes up with good new ideas,” “new ideas from our team are always useful,” “our team is creative,” and “our team’s new ideas are useful to the team.” (Cronbach’s *α* = 0.790).

#### 3.2.5. Control variables

We controlled for sex, age and education at the individual level.

## 4. Results

AMOS software (version 21.0) was used to conduct confirmatory factor analysis of the variables (Table 1). Table 1 shows that the four-factor model fitting index generated the optimal result (Δ chi-square = 749.65, Δdf = 3, *p* < 0.001)—implying that the model was superior to other alternatives. None of the other model fitting indicators reached a significant value, indicating that the measurement model had good discriminative validity.

**Table 1:** The results of the confirmatory factor analysis.

Model	*χ* ^2^	df	*χ*^2^/df	CFI	TLI	RMSEA	SRMR	Δ*χ*^2^
Four factor model (A, B, C, D)	767.517	318	2.414	0.932	0.925	0.054	0.056	
Three factor model (A, B, C + D)	1517.17	321	4.726	0.82	0.803	0.088	0.14	749.65
Two factor model (A + C + D, B)	1543.22	323	4.778	0.816	0.8	0.088	0.14	26.05
One factor model (A + B + C + D)	1570.38	324	4.847	0.812	0.796	0.089	0.141	27.16

A, Sense of work gain; B, LMX; C, Work smoothness; D, Team creativity.

Using SPSS 22.0 and Harman’s method, each questionnaire was used as all the items in the exploratory factor analysis, indicating that there was no serious common method deviation in the present study. The results showed that the characteristic roots of the three factors were greater than 1, and 38.84% of the variance was explained by the first common factor (which was less than 40%). No significant common method variance was observed.

The means, standard deviations, and correlations for each variable are presented in Table 2. There was a significant positive correlation between sense of work gain and team creativity (*r* = 0.786, *p* < 0.01). Sense of work gain was positively related to LMX (*r* = 0.831, *p* < 0.01), and LMX was positively related to team creativity (*r* = 0.751, *p* < 0.01). The analysis provides the necessary prerequisite for further exploration of the relationship between the variables in this study.

**Table 2:** Means, SD, and correlations of the variables.

Variables	*M*	SD	1	2	3	4	5	6	7	8
1. Sex	1.58	0.49								
2. Age	30.13	5.32	−0.140^**^							
3. Education	2.62	0.75	0.028	−0.07						
4. Length of service	2.56	0.99	−0.113^*^	0.342^**^	0.027					
5. Work smoothness	9.65	3.89	0.170^**^	−0.118^**^	0.012	−0.202^**^				
6. Sense of work gain	50.31	10.11	0.032	−0.263^**^	0.315^**^	−0.064	0.130^**^			
7. LMX	26.58	5.86	0.031	−0.246^**^	0.300^**^	−0.085	0.091^*^	0.831^**^		
8. Team creativity	15.45	3.39	0.004	−0.316^**^	0.278^**^	−0.093^*^	0.095^*^	0.786^**^	0.751^**^	

*n* = 484, *p < 0.05, **p < 0.01.

As shown in Table 2, educational background was significantly and positively correlated with sense of job gain, LMX, and team creativity. Age had a significant negative correlation with job smoothness, sense of job gain, LMX, and team creativity. Therefore, it was necessary to control for these demographic variables.

The data were processed using Progress. The hypotheses results, after controlling for gender, age, and education, are outlined in the following paragraphs.

Model 1 in Table 3 shows that sense of work gain positively predicted team creativity (*β* = 0.74, *p* < 0.01), which supports Hypothesis 1. Model 2 shows that LMX has a positive predictive effect on team creativity (*β* = 0.31, *p* < 0.01). Thus, Hypothesis 2 was supported.

**Table 3:** Variables regression analysis.

Variables	Team creativity					
	Model 1		Model 2		Model 3	
	*β*	*t*	*β*	*t*	*β*	*t*
Intercept	0.96	2.89^**^	0.1	0.67	0.06	0.40
Control Variables
Sex	−0.08	−1.41	−0.06	−1.02	−0.05	−0.1
Age	−0.23	−4.25	0.03	0.83	0.05	1.27
Educational	0.05	1.30	−0.04	−1.38	−0.04	−1.47
Sense of work gain	0.74	24.40^**^	0.52	10.59^**^	0.49	10.03^**^
Work smoothness					0.02	0.55
LMX			0.31	6.34^**^	0.33	6.83^**^
SWG*WS					0.14	3.13^**^
LMX*WS					−0.2	−4.59^**^
R-sq	0.63^**^		0.31^**^		0.67^**^	
F	204.07		271.58		118.17	

SWG, Sense of work gain; WS, Work smoothness; *p < 0.05, **p < 0.01.

As shown in Table 4, the mediating influence of LMX between sense of work gain and team creativity was 0.25, with a confidence interval of (0.11, 0.38), excluding 0. Therefore, Hypothesis 3 was verified.

**Table 4:** Bootstrap results.

Pathway	Standardized indirect effects	95% Confidence interval	
		Lower bounds	Upper bounds
SWG-LMX-TC	0.25^**^	0.11	0.38

SWG, Sense of work gain; WS, Work smoothness; TC, Team creativity; *p < 0.05, **p < 0.01.

**Figure 1:**
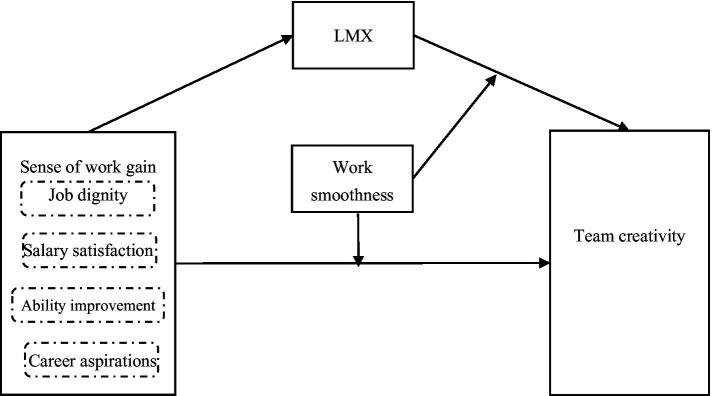
Theoretical model.

To better test the existence of the moderating effect of Model 4, we combined the process data and plotted the results in Table 5. The interaction between sense of work gain and work smoothness had a moderating effect on the SD + 1, SD, and SD-1 paths, while that between LMX and work smoothness had a significant moderating effect on the SD + 1 and SD paths only. Tables 2, 3 present the moderating effects (Figure 1).

**Table 5:** Test for moderating effects.

Interaction items	Moderating effects		95% Confidence interval	
			Lower bounds	Upper bounds
SWG*WS	SD + 1	0.32	0.16	0.48
	SD	0.47	0.37	0.57
	SD-1	0.69	0.55	0.84
LMX*WS	SD + 1	0.57	0.43	0.72
	SD	0.36	0.27	0.46
	SD-1	0.05	−0.09	0.16

SWG, Sense of work gain; WS, Work smoothness; TC, Team creativity.

**Figure 2:**
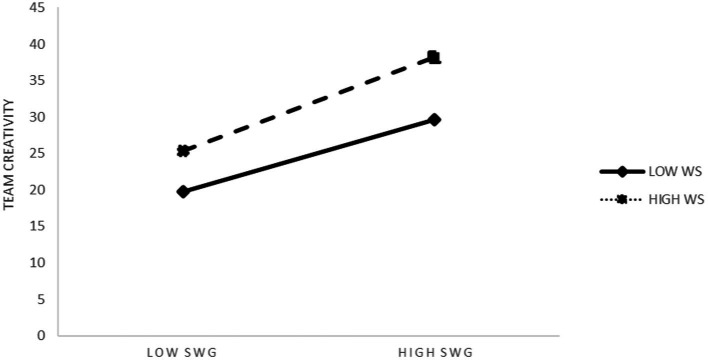
Work smoothness plays a role in regulating the sense of work gain and team creativity. SWG, Sense of work gain; WS, Work smoothness; LMX, Leader-member exchange.

The results of the simple slope analysis (Model 3 of Table 3) to verify adjustment for work smoothness show that the interactions between sense of work gain and work smoothness predicted changes in team creativity. As Figure 2 shows, work smoothness enhanced the impact of sense of work gain on team creativity, and the different levels of work smoothness verified the significance of its adjustment role. Thus, Hypothesis 4 was supported.

**Figure 3:**
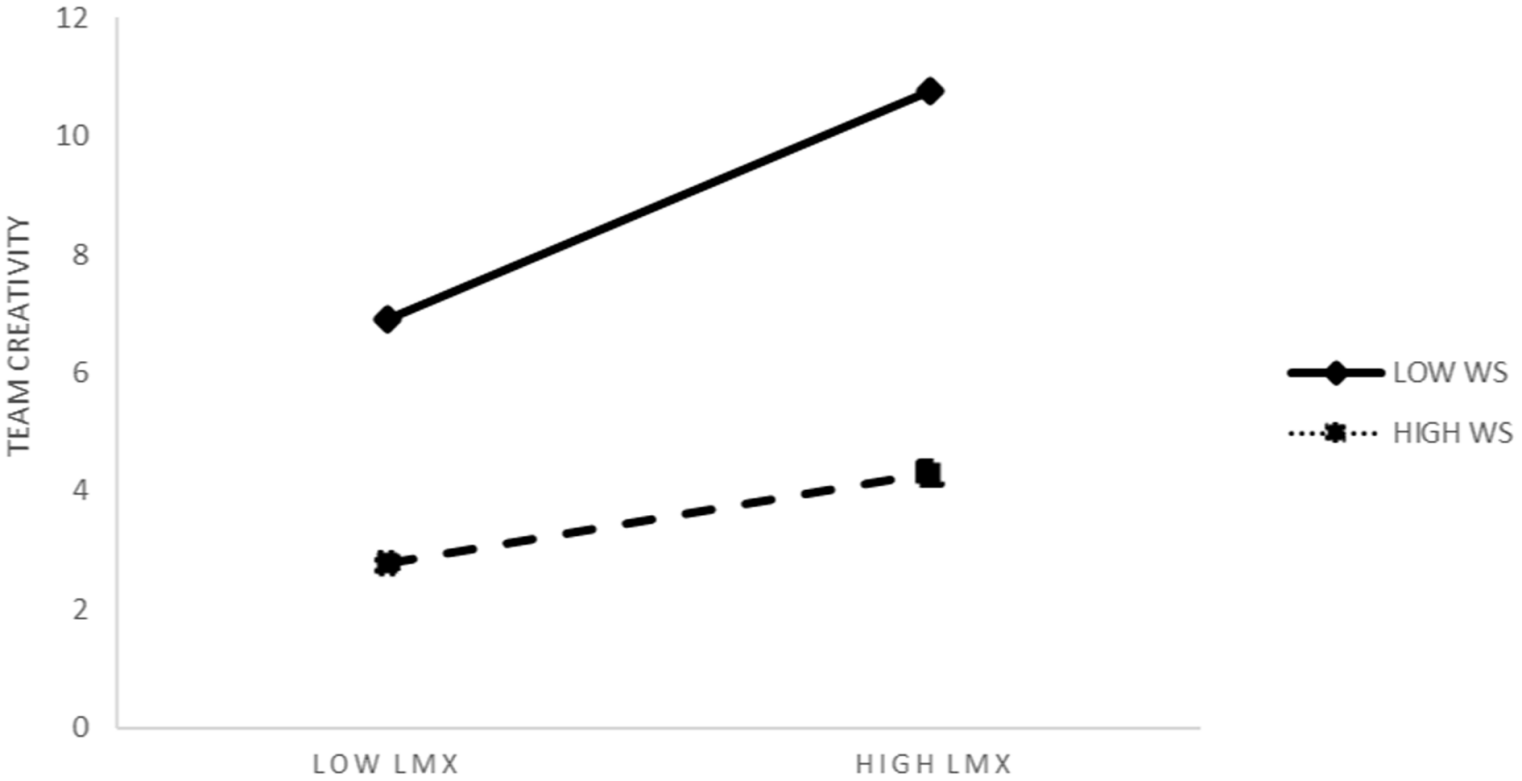
Work smoothness plays a role in regulating LMX between team creativity. LMX, Leader-member exchange; WS, Work smoothness.

The simple slope analysis (Model 3 of Table 3) to verify adjustment for work smoothness also revealed that LMX and work smoothness interaction items negatively predicted team creativity. Thus, Hypothesis 5 was supported. As Figure 3 shows, work smoothness enhances LMX’s impact on team creativity. Different levels of work smoothness demonstrated significant moderation. Interestingly, work smoothness moderated the mediating effect of LMX on team creativity, such that the mediating effect was stronger when work smoothness was lower.

## 5. Discussion

As per our proposed hypotheses, after controlling for gender, age, education, and length of service, the analysis results indicated sense of work gain positively predicts team creativity in two ways. First, LMX plays a mediating role between sense of work gain and team creativity, and LMX positively predicts team creativity. Second, work smoothness, as a moderating variable, positively regulates the relationship between sense of work gain and team creativity. The research also found that, contrary to the hypothesis, work smoothness negatively moderates the relationship between LMX and team creativity. Compared with a high level of work smoothness, a low level of work smoothness had a stronger effect on the relationship between LMX and creativity.

However, the above analysis raises the following question: within a team, how do employees perceive how LMX and LMX differentiation affects themselves? LMX can be broadly divided into three phases. The initial phase is when newcomers enter the organization (Dansereau et al., 1975) and the leadership evaluates their skills or abilities and supports their potential. The second phase involves role-building LMX, represented by negotiation and trust building (Graen et al., 1982), which facilitates the formation of a more comfortable relationship between leaders and employees as they slowly move through their roles. The third phase is established based on maintenance—the quality of LMX between the leader and employee gradually stabilizes, and the differences between members become increasingly apparent (Leah, 2013). One purpose of this study was to explore the impact of LMX on team creativity. This discussion is based on the first and second phases of LMX, highlighting the unique role of employees’ sense of work gain and work smoothness in an atmosphere of organizational trust and harmony.

This study proposes that the reason for the aforementioned situation is that, in the dynamic development of leaders and members, employees share the same work environment, similar cognitive levels, and unified work patterns, which are prone to phenomena such as group thinking and group polarization (Chen, 2021). An increase in work smoothness exacerbates this familiarity, leading to lower team creativity. Lower work smoothness means that employees have different thought processes—making it easier to generate ideological communication and conflict. As a result, team creativity at low levels of work smoothness is higher than that at high levels of work smoothness.

### 5.1. Theoretical contribution

This study explored and confirmed that job acquisition can be an antecedent variable of team creativity. We included work smoothness in the study and further explored the mediating mechanism and boundary conditions of team creativity in relation to sense of work gain. As a new concept, sense of work gain was proposed in conjunction with the Chinese enterprise context, and combined with empirical research data to deepen our understanding of the mechanism of the antecedent variables of team creativity.

Specifically, although existing studies confirm the positive impact of sense of work gain on corporate employees, this study builds a theoretical bridge between sense of work gain and team creativity by means of empirical research to expand the findings on antecedent variables of team creativity. Second, we explored the mediating mechanism in conjunction with LMX, based on the premise that sense of work gain is a positive psychological experience reflecting employees’ workplaces and can form a separate construct (Zhu and Liu, 2020). Based on this premise, we integrated the findings of positive psychology and psychological contract theory to build a mediating mechanism between sense of work gain and team creativity with LMX. Finally, work smoothness was measured by reverse scoring of work detachment, focusing on the moderating role of positive emotions in the enterprise; further, the positive emotion expansion theory was confirmed in terms of management practice.

### 5.2. Practical implications

For leaders and HR professionals looking to increase employee initiative and motivation, our research shows that employees’ access to work gains can impact their creativity.

First, the higher the employee’s sense of work gain, the greater the development of their self-efficacy at work. This is more conducive to realizing their own value, affecting employees’ job well-being and organizing civic behavior (Zhu and Liu, 2020), thus stimulating team creativity. Therefore, managers should pay more attention to employees’ work experience besides their work output.

Second, LMX plays an intermediary role between sense of work gain and team creativity. On the one hand, it is suggested that managers should pay attention to communication with employees and cultivate high-quality LMX relationships, so that subordinates can experience more emotional exchange and enhance their sense of belonging. On the other hand, managers should ensure that “insiders” and “outsiders” should not be separated within the team; they should instead create a harmonious atmosphere within the team instead of fostering internal competition. However, in an actual work environment, it is important to consider that employees have different experiences at different stages of LMX. In the dynamic development of leaders and members, employees share to phenomena such as group thinking and group polarization ((Chen, 2021) that has negative effects on employee creativity.

Finally, examining the adjusted model of work smoothness showed that when employees get help from leaders and colleagues to make work smoother, employee productivity can greatly improve. Managers in the enterprise should establish a mutually supportive working atmosphere to enhance team creativity. Although work smoothness improvement is not always positive, a high level of work smoothness means that employees’ sense of work gain can improve team creativity. LMX had a more significant impact on team creativity at a lower level of work smoothness. Considering these findings, we recommend that managers integrate reasonable resources into the work environment for teams.

### 5.3. Research limitations and prospects

There are some limitations to this study. First, this study used a cross-sectional approach—that is, variables were collected at the same time point. However, the study of variables is a dynamic development process, and cross-sectional research is insufficient to strictly reflect the causal relationship between variables. Future research should use point-in-time measurements or vertical tracking methods for more rigorous testing. Second, the concept of sense of work gain is a native concept in China and the respondents belonged to Chinese companies. Future studies should consider cross-cultural research to explore the role of cultural and social background.

## 6. Conclusion

This study reveals how sense of work gain promotes team creativity through the mediating variable LMX and the moderating variable work smoothness. The results show that LMX can positively predict team creativity. Work smoothness moderates the relationship between sense of work gain and team creativity in two ways: (a) it can effectively enhance the direct impact of a sense of work gain on team creativity and (b) it moderates the relationship between LMX and team creativity to influence the mediating role of LMX between sense of work gain and team creativity. Furthermore, we found that the different levels of work smoothness have significantly varying influence on the relationship between LMX and team creativity.

## Data availability statement

The raw data supporting the conclusions of this article will be made available by the authors, without undue reservation.

## Ethics statement

The studies involving human participants were reviewed and approved by Ethics Committee of Department of Psychology, Southwest University. Written informed consent for participation was not required for this study in accordance with the national legislation and the institutional requirements.

## Author contributions

HZ and KS conceived and designed the experiment and reviewed and edited the paper. KS performed the experiment. HZ, KS, and ZZ analyzed and discussed the data and wrote the original draft. All authors contributed to the article and approved the submitted version.

## Conflict of interest

The authors declare that the research was conducted in the absence of any commercial or financial relationships that could be construed as a potential conflict of interest.

## Publisher’s note

All claims expressed in this article are solely those of the authors and do not necessarily represent those of their affiliated organizations, or those of the publisher, the editors and the reviewers. Any product that may be evaluated in this article, or claim that may be made by its manufacturer, is not guaranteed or endorsed by the publisher.

## Appendix

My work is affirmed by superiors.

My work is recognized by colleagues.

The organization recognizs my work achievements.

My salary is reasonable.

I get the desired salary.

My workload is relatively fair.

My work enables me to master the job knowledge and skills.

My work improves my ability to analyze problems.

My work enhances my competence in solving work problems.

My work improves my ability to cooperate with others.

My work is related to my career ideal.

I have full confidence in my development in the organization.

I am full of hope for my future career development.*
